# Trauma-informed interventions versus control for cancer-risk behaviours among adults: rationale and design for a randomized trial

**DOI:** 10.1186/s12889-019-7641-0

**Published:** 2019-10-29

**Authors:** Cheryl L. Currie, Jennifer L. Copeland, M. Lauren Voss, Lisa-Marie Swanepoel, Mirela Ambeskovic, Nimesh B. Patel, Erin K. Higa

**Affiliations:** 1 Faculty of Health Sciences, University of Lethbridge, M3083 Markin Hall, 4401 University Drive, Lethbridge, AB T1K 3M4 Canada; 2 Department of Kinesiology and Physical Education, University of Lethbridge, Lethbridge, Canada; 3 Canadian Centre for Behavioural Neuroscience, University of Lethbridge, Lethbridge, Canada

**Keywords:** Cancer-risk behaviour, Smoking, Alcohol, Yoga, Drumming, Psychoeducation

## Abstract

**Background:**

Tobacco use, alcohol use, and sugar-sweetened beverage consumption are each associated with increased cancer-risk. Psychological trauma is a common experience and a key driver of these behaviours among adults. The primary aim of this study is to evaluate the effect of trauma-informed yoga, drumming, and psychoeducation compared to control on tobacco use, alcohol use, and sugar-sweetened beverage consumption among community-based adults. Secondary aims are to evaluate the effect of these interventions compared to control on psychological and physiological stress symptomology, social connection, and coping behaviour.

**Methods:**

Recruitment for this single-blinded randomized trial began in April 2019 in the Faculty of Health Sciences at the University of Lethbridge. Adults who consumed tobacco, alcohol, or sugar-sweetened beverages in the past month and live in Lethbridge, Alberta are being recruited using ads placed in public spaces. Participants are randomly allocated to a 12-session group yoga class, 12-session group drumming class, a 12-session psychoeducation class, or control. Participants attend an appointment in-person to fill out an online questionnaire package, provide a saliva sample, and complete physical measures pre-intervention, and 1-month and 6-months post-intervention.

**Discussion:**

This study provides a unique opportunity to compare the impacts of two trauma-informed body-based interventions to psychoeducation and control for cancer-risk behaviour among community-based adults. The findings can be used to develop trauma-informed group interventions to reduce cancer-risk behaviour in general populations. Results are expected in 2022.

**Trial registration:**

This trial was registered with ClinicalTrials.gov ISRCTN15583681 on 22 August 2019 (retrospectively registered).

## Background

Well established cancer-risk behaviours include smoking and alcohol use. The association between tobacco use and the development of various cancers is strong, dose-dependent and insensitive to covariate-adjustment, with systematic reviews demonstrating that smoking considerably enhances and cessation delays the risk of developing and dying of cancer [1–5]. Causal relationships between alcohol consumption and cancer of the oral cavity, pharynx, larynx, esophagus, liver, colorectum and breast are well established, with relative risks rising in a dose-response fashion with increasing alcohol consumption for all named sites [6–10]. There is no safe threshold of alcohol intake for cancer risk [9, 11, 12]. Alcohol is a major contributor to cancer mortality and years of potential life lost due to cancer. The habitual consumption of sugar-sweetened beverages is an emerging risk factor for cancer associated with the increased incidence of pancreatic, gallbladder and biliary tract cancer; with cancer reoccurrence and mortality generally; and with important risk factors for cancer (e.g., type 2 diabetes, obesity) [13–17].

Much of the effort to prevent cancer-risk behaviours have been based on the theory that lack of knowledge is a key driver. Thus, mass media campaigns have been a central focus for prevention efforts [18]. Yet, despite the enormous amount of resources invested, a recent systematic review of reviews found limited evidence that mass media campaigns have an impact on alcohol use and diet, and mixed evidence for impacts on tobacco use [19]. To make progress, there is a need to move beyond the assumption that cancer-risk behaviours are wholly determined by *individual susceptibility* due to lack of knowledge. We must also consider *population susceptibility* due to common agents present within the social milieu in which we grow, live, work and age [20].

### Social trauma and cancer-risk behaviour

Humans are naturally inclined to establish and maintain profound bonds within society [21]. Factors that interfere with this natural process such as adverse childhood experiences (ACEs) have been shown to increase cancer-risk behaviour. A review of 155 quantitative, peer-reviewed US studies indicates adults will use the psychoactive properties of nicotine and alcohol, or may engage in emotional eating and/or excessive sugar consumption to manage the dysphoria associated with ACEs [22]. ACEs include physical, emotional and sexual abuse; emotional and physical neglect; and/or household dysfunction experienced before 18 years of age (i.e., domestic violence, mental illness and addiction in the household, parental separation and parental incarceration) [23].

In North America exposure to ACEs is common with the majority of adults reporting at least one ACE (52–67%), two ACEs (26–42%), and up to as many as four or more ACEs (6–16%) by the age of 18 years [23–28]. In Canada, the prevalence of ACEs and other forms of social trauma may be further elevated among Indigenous adults given child maltreatment was a common experience in residential school up until the last school closed in 1996, and Indigenous adults also report high levels of racial discrimination and social exclusion across the life course [29–32].

### Interventions

In North America, there is a strong focus on the verbal narrative in psychological and psychiatric treatment. Yet the rational, executive brain has been shown to have limited capacity to control emotional and physiological arousal in response to trauma triggers [33]. Specifically, the autonomic nervous system interferes with executive function once sensory triggers of past trauma activate the brain to engage in habitual, self-protective behaviour [34]. Compounding this problem, decreased activation of the medial prefrontal cortex among individuals who have experienced trauma makes it more difficult to become aware of these internal states, and when they are being activated [35]. Further, many traumatized individuals have also learned to disassociate from the body as a form of self-protection [36, 37]. Thus, it may be more difficult to remain aware of, or concerned about, the ways in which tobacco use, alcohol use, and sugar-sweetened beverage consumption are affecting the physical body. Body-oriented interventions designed to increase awareness of physical sensations, muscle activation, and the movement of the body may offer the opportunity to reprogram automatic physiologic hyperarousal in response to triggers, while at the same time increasing positive body awareness, and mindful attention to the ways in which various habitual self-protective behaviours may be impacting physical health.

Given most traumatic experiences occur in the context of interpersonal relationships, the resulting boundary violations and loss of autonomous action can interfere with the ability to form trusting relationships with others [38]. Alexander has posited that such experiences result in *social dislocation*, defined as an enduring lack of psychosocial integration in society, an experience that is both individually painful and socially destructive [21]. From this perspective, engaging in cancer-risk behaviour may be a way of adapting to the discomfort of sustained social dislocation by providing the rewards an individual would normally receive through their social world. Group interventions designed to build social trust may be effective in reducing these behaviours.

The premise for this study is that adults in Canada are susceptible to cancer-risk behaviour due to the pervasiveness of ACEs and other forms of social trauma within society, and that addressing the psychological symptomology, physiologic sequelae, and social dislocation associated with interpersonal trauma may reduce cancer-risk behaviour and increase healthy coping behaviour. We will examine this premise by comparing three 12-session interventions that have been designed to emphasize the connection between mind and body and build group trust, to control on three primary endpoints (tobacco use, alcohol use, and sugar-sweetened beverage consumption). We will also examine changes in four secondary endpoints (psychological stress symptomology, physiological stress symptomology, social dislocation, and coping behaviour choices).

Trauma-informed yoga. Twelve group yoga sessions, delivered weekly, will use breathing exercises, physical postures, and mindfulness meditation to direct participant attention toward internal states and the connection between mind and body [39]. Yoga has been shown to impact both psychological and physiological sequelae associated with trauma [40–42]. It has been theorized that trauma-informed yoga (TIY) that emphasizes choice, as well as curiosity about bodily sensations may strengthen these impacts [39, 43]. In the present study, licensed yoga instructors with TIY training will guide participants using language modified to reduce the likelihood it may trigger traumatic memories. Invitatory language will encourage participants to make choices (e.g., when you are ready, I invite you to experiment with lifting your arms), and reflect on how the movements and breathing they choose to engage in resonates within their body (e.g., you might consider what it feels like to stretch your calf in this way).

Trauma-informed drumming. Twelve group drumming sessions, delivered weekly, will offer a patterned, repetitive, rhythmic experience that can be effective in regulating the brainstem and neural network among adults who have experienced trauma. At a psychological level, group drumming has been shown to improve mood and reduce trauma-related symptoms including PTSD, anxiety, and impulsivity; and promote self-expression, social cohesion, and engagement in treatment [44–46]. At a physiologic level, group drumming that emphasizes camaraderie, group acceptance, and nonjudgmental performance has been shown to attenuate physiological stress response patterns, resulting in statistically significant increases in plasma dehydroepiandrosterone-to-cortisol ratios, natural killer cell activity, lymphocyte-activated killer cell activity; as well as improve pain tolerance; and decreased blood pressure and inflammation [47–52]. In this study, group drumming will be delivered using trauma-informed, invitatory language. Participants will be invited to engage various drums and rhythms, and to reflect on how these sensations resonate within their body.

Trauma-informed psychoeducation. Twelve group psychoeducation sessions, delivered weekly, will be delivered using trauma-informed, invitatory language. Licensed counsellors will teach adults about various forms of social trauma, how these experiences impact the body and mind, and ways to respond to and cope with stress in healthy ways.

## Methods/design

### Objectives

The primary objective is to evaluate the effect of trauma-informed group yoga, drumming, and psychoeducation compared to one another and control on tobacco use, alcohol use, and sugar-sweetened beverage consumption among community-based adults.

Secondary objectives are to evaluate the effect of trauma-informed group yoga, drumming, and psychoeducation compared to one another and control on several outcomes including: (1) psychological stress symptomology, (2) physiologic stress symptomology, (3) social dislocation, and (4) health coping behaviour among community-based adults.

### Trial design and study setting

The CRIS (Cancer-Risk Interventions Study) trial is a parallel, 4-arm randomized controlled trial with blinding of assessors. The study was approved by the Human Research Ethics Board of Alberta Cancer Committee. The trial started in April 2019 led by Dr. Cheryl Currie in the Faculty of Health Sciences at the University of Lethbridge in Alberta, Canada. The trial is expected to end in late 2021. Participation in each condition will last approximately 44 weeks (8 weeks prior to the intervention to complete baseline data collection with all participants, 12 weeks for the intervention, and 24 weeks post-intervention). A schedule of the CRIS trial is provided in Table 1.

**Table 1:** Participant timeline

Item	Instrument	Study Period				
		Enrollment	Baseline Assessment/ Allocation	Post-Allocation 12-Week Intervention	Follow-up 1 month	Follow-up 6 months
		T_0_	T_1_	T_2_	F_1_	F_2_
Eligibility screen	N/A	X				
Informed consent	N/A		X			
Allocation	N/A		X			
Primary outcomes						
1. Smoking	4 items; salivary cotinine		X		X	X
2. Alcohol use	AUDIT		X		X	X
3. Sugar beverage use	3 items		X		X	X
Secondary outcomes						
1. *Psychological stress symptomology*						
PTSD	PTSD Checklist		X		X	X
Dissociative experiences	BDE-S		X		X	X
Depression	2 items		X		X	X
Suicide	2 items		X		X	X
Complicated grief	BGQ		X		X	X
Self-esteem	RSES		X		X	X
Self-compassion	SCS-SF		X		X	X
Resilience	CD-RISC		X		X	X
2. *Physiologic stress symptomology*						
Neuroendocrine	Salivary DHEA-S		X		X	X
Metabolic	BMI, waist circumference		X		X	X
Cardiovascular	Blood pressure, heart rate, uric acid		X		X	X
Immune	Salivary markers: IgA, CRP, Interleukins IL-1β, IL-6, IL-8, TNF-α		X		X	X
General health	14 items				X^a^	X^a^
3. *Social dislocation*						
Social support	SSQ		X		X	X
Community connectedness	VI		X		X	X
4. *Coping behaviour*						
Physical activity	IPAQ		X		X	X
Sedentary behaviour	IPAQ				X	X
Sleep behaviour	PSQI		X		X	X
Eating habits	7 items		X		X	X
Drug use /dependence	DUDIT		X		X	X
Covariates						
1. *Social trauma*						
Childhood adversity	ACE Survey		X			
Racial discrimination	EOD Scale		X			
Indigenous residential school attendance	4 items		X			
Foster care/adoption	2 items		X			
Domestic violence	5 items		X			
Historical loss	HLS		X			
2. *Sociodemographics*			X		X	X
Group Interventions						
Yoga						
Drumming				X		
Psychoeducation				X		
Control				X		

^a^Health questions relevant for salivary measures will be asked at F_1_ and F_2_ (e.g., pregnancy, having a cold or flu, dental work/care). T_0_, baseline; T_1_, intervention allocation; T_2_, 12 week intervention for those not randomized to control; F_1_, first follow up appointment 1 month after intervention completion; F_2_, second follow up appointment 6 months after intervention completion; *AUDIT* Alcohol Use Disorders Test, *DUDIT* Drug Use Disorders Test, *IPAQ* International Physical Activity Questionnaire – Short Form, *HPA Axis* Hypothalamic Pituitary Adrenal Axis, *DHEA-S* Dehydroepiandrosterone sulfate, *BDE-S* Brief Dissociative Experiences Scale, *RSES* Rosenberg Self-esteem Scale, *SCS-SF* Self-compassion scale – short form, *PSQI* Pittsburgh Sleep Quality Index, *CRP* C-Reactive Protein, *BMI* Body Mass Index, *ACEs* Adverse Childhood Experiences Scale, *EOD* Experiences of Discrimination Scale, *BGQ* Brief Grief Questionnaire, *SSQ* Social Support Questionnaire, *HLS* Historical Loss Scale, *VI* Vancouver Index

### Participant and public involvement

Over an 18-month period beginning in June 2017 and ending December 2018, we received input from mental health professionals, members of the public, and Indigenous Elders and stakeholders who have expertise in and/or have struggled with psychological trauma or cancer-risk behaviour. Based on these consultations we will oversample Indigenous adults for each study arm and interventions will be run separately for males and females. Participants will also be provided the opportunity to have their saliva samples returned to them or included in a ceremony led by an Indigenous Elder to return them to the Earth, in keeping with Indigenous cultural protocols in our territory.

### Eligibility criteria

**Fig. 1:**
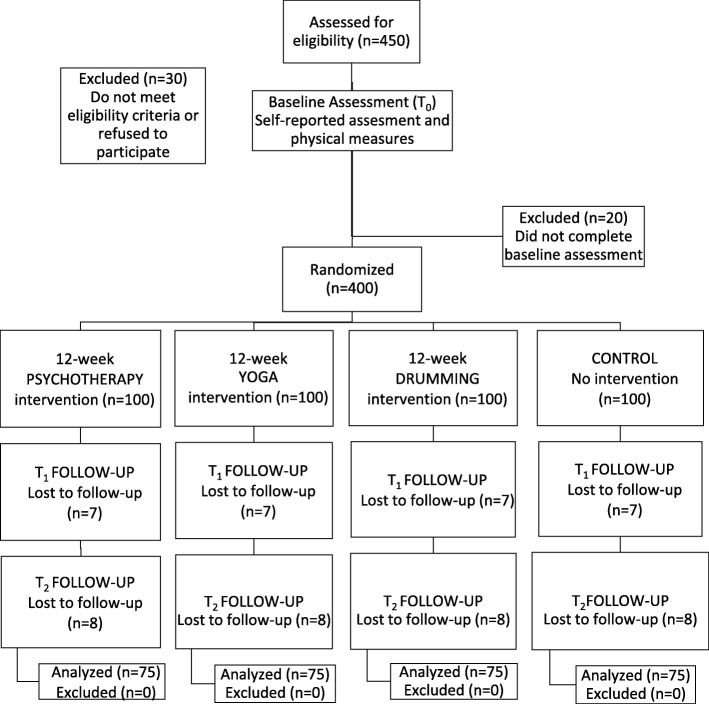
Study flowchart

The study is recruiting a community-based sample of 400 adults that meet eligibility criteria to achieve 300 participants (approximately 25% loss-to-follow-up, *n* = 75 per arm, see Fig. 1).

#### Inclusion criteria

Participants must satisfy the following criteria to be enrolled in the study:

- Aged 18 and over;
- Used a nicotine-containing substance, consumed alcohol, or consumed a sugar-sweetened beverage at least once *in the past month*;
- Live at a permanent address in the target city; and
- Plan to live in the target city for the next 12 months.

#### Exclusion criteria

Participants meeting any of the following criteria will be excluded from the study:

- Unwilling to give consent
- Lacking capacity to give consent

### Participant screening, recruitment and consent

Participants are being recruited using posters placed in public spaces. We seek to oversample Indigenous adults so that stratified analyses can take place, thus some ads are specifically being placed within Indigenous organizations and agencies in the target city. Those interested are asked to contact the study coordinator. Screening and study enrollment is conducted during this phone call, text or email communication. Those eligible are scheduled to attend an appointment with two research team members (i.e., assessors) to complete informed consent and baseline measures (expected mean completion time = 75 min) during standard office hours (9:00 am – 4:00 pm). To strengthen enrollment, improve adherence and reduce loss-to-follow-up participants will receive a $25 gift card for attending this appointment, and all subsequent components of the study they take part in (e.g., each intervention session and data collection appointment they attend).

### Assignment of interventions

Unassigned participant IDs were randomly allocated by two team members (LV, LS) to one of four conditions generated using permuted blocks of four using https://www.randomizer.org/. The intervention that an ID was assigned was placed in a sealed envelope and kept in the main study lab. Participant IDs are being assigned consecutively as participants are enrolled. When the participant leaves the data collection appointment, they receive the envelope with information on the intervention they have been assigned including the date, time and location; and contact information for the coordinator leading the intervention they have been assigned to, who will send them a reminder before the intervention begins.

Assessors remain blinded to the allocation of the participant during baseline data collection. Participants are asked not to communicate with the assessors about the intervention received. At the two remaining data collection timepoints we will examine the success of assessor blinding by asking whether the assessor thought the participant had been allocated to a specific arm of the trial, including the percentage of certainty. (i.e., 50% would be interpreted as a pure guess). No project team member can change the group a participant has been assigned to. Team members performing statistical analyses will also be blinded to the allocation of participants.

### Interventions

Participants will engage in one of four conditions. For each intervention arm, weekly sessions will take approximately 90–120 min. All spaces selected for interventions are comparable in terms of room accessibility, size, natural light, and configuration.

#### Trauma-informed yoga

Group yoga will be delivered once a week for 12 weeks by two licensed yoga instructors who have completed training in trauma-informed yoga delivery (approximately 25 participants per class). Each session will begin with an invitation to sit in a semi-circle facing the instructors on a yoga mat or chair placed behind a mat. Participants can engage in the class from the mat or chair or move between them as the class unfolds. One instructor will lead the class from the mat, while the second will silently demonstrate the same practice using the chair. The instructor will begin the class by introducing the week’s theme (e.g., grounding and safety, centering, non-attachment, imprints of the past, connection to nature) (10 min), followed by a breath practice (10 min), a yoga practice (50 min), a meditation practice (10 min), and closing words (10 min).

#### Trauma-informed drumming

Group drumming will be delivered once a week for 12 weeks by two instructors certified to deliver the Integrative Drum Circles (IDC) method (approximately 25 participants per class) [53, 54]. Each session will begin with an invitation to sit on a chair beside a drum arranged in a semi-circle facing the instructors. One instructor will lead the class, while the second instructor will silently demonstrate the same practice for participants. An instructor will begin the class by introducing the week’s theme (e.g., respect, gratefulness, gentleness) (10 min), followed by a drum practice using the IDC method (70 min), and closing words (10 min).

#### Trauma-informed psychoeducation

Group psychoeducation will be delivered once a week for 12 weeks by two licensed and experienced counsellors (approximately 12–13 participants per class, with two separate sessions running each week as 25 is too many to accommodate at one time for this intervention). Each session will begin with an invitation to sit on a chair arranged in a semi-circle facing the facilitators. One facilitator will lead the session each week. The facilitator will begin the class by introducing the week’s theme (e.g., setting goals, being mindful) (10 min), followed by a lecture and activities to integrate learning (e.g., group discussion, journaling) (70 min), and closing words (10 min).

#### Control

The control group will not receive an intervention.

### Criteria for discontinuation

Participants who miss 50% of the intervention they are assigned to will meet criteria for discontinuation of the trial.

### Fidelity

The extent to which each intervention adheres to the program model developed for it will be assessed by research staff present at the session who will record the amount of program content delivered for a specific session, adherence to program content, and the quality of delivery. Facilitators will also be asked to reflect on program fidelity for a given session when it ends using a prepared checklist with space for open-ended reflection.

### Outcomes

Primary and secondary outcomes will be assessed at baseline, within 1-month post-intervention, and again 6 months post-intervention. The three primary outcomes are smoking, alcohol and sugar-sweetened beverage use. *Smoking* will be assessed by a change in the number of cigarettes smoked per day or per week, as measured by self-report, and changes in salivary cotinine which is a biochemical measure of active smoking and smokeless tobacco use [55]. Saliva samples will be collected during the first office visit at 3 time points using the passive drool technique. Participants will be asked to rinse their mouth with bottled water upon arrival and the first sample will be collected after completion of the demographic portion of the questionnaire. Remaining samples will be taken 30 and 60 min after questionnaire completion. Whole saliva samples will be collected in a 2 ml microcentrifuge tube using a Saliva Collection Aid (Salimetrics, State College, PA). During data collection, salivary samples will be stored on dry ice in a cooler and then transferred immediately to a lab where samples were stored at − 80 °C until analysis. Anonymized salivary data will be sent to Salimetrics in Carlsbad, CA for analysis, then returned.

*Alcohol use* will be assessed by a change in the frequency of alcohol use and hazardous alcohol use using the Alcohol Use Disorders Identification Test (AUDIT), a 10-item self-report measure developed by the World Health Organization for detecting alcohol use problems [56, 57]. *Sugar-sweetened drink consumption* will be assessed by a change in how frequently participants consume beverages sweetened with sugar, honey or syrup using self-report questions. Secondary outcomes are psychological stress symptomology, physiologic stress symptomology, social dislocation, and coping behaviour, which will be examined using the constructs and measures outlined in Tables 1 and 2.

**Table 2:** Study biomarkers

Biomarkers	Source	Function	Measurement Procedure	Analysis
1. Immune				
CRP	Saliva	Inflammatory marker.	Passive drool method^a^	Lab analysis^b^
Secretory IgA	Saliva	Immune function of mucous membranes.	Passive drool	Lab analysis
IL-1β	Saliva	Pro-inflammatory cytokine	Passive drool	Lab analysis
IL-6	Saliva	Pro- and anti-inflammatory cytokine	Passive drool	Lab analysis
IL-8	Saliva	Pro-inflammatory cytokine	Passive drool	Lab analysis
TNF α	Saliva	Pro-inflammatory cytokine	Passive drool	Lab analysis
2. *Neuroendocrine*				
DHEA-S	Saliva	Stress-response marker	Passive drool	Lab analysis
3. Metabolic				
BMI		Health-risk indicator for cardiometabolic disease	Height and weight will be measured once, to the nearest 0.5 cm and 0.1 kg, using a Health O Meter mechanical beam scale and stadiometer.	Participant’s weight will be divided by their height.
WC	Iliac crest	Health-risk indicator for cardiometabolic disease	WC will be measured twice at the top of the iliac crest, to the nearest 0.5 cm.	The two measures will be averaged.
4. Cardiovascular				
BP	Brachial artery	Pressure of blood in the arteries	Resting systolic and diastolic blood pressure will be measured using a Life Source automated sphygmomanometer. Participants will be seated and resting quietly for 3 measurements.	The first reading will be discarded. The second and third readings will be averaged.
HR	Brachial Pulse	Estimate of cardiovascular fitness and heart function	Resting heart rate will be measured using a Life Source automated sphygmomanometer. Participants will be seated and resting quietly for 3 measurements.	The first reading will be discarded. The second and third readings will be averaged.
Uric Acid	Saliva	Associated with cardiovascular responses to stress	Passive drool	Lab analysis
Tobacco Use				
Cotinine	Saliva	Biomarker for exposure to tobacco smoke	Passive drool	Lab analysis

*CRP* C-reactive protein, *IL-1 Beta* Interleukin-1 Beta, *IL-6* Interleukin-6, *IL-8* Interleukin-8, *TNF α* Tumor necrosis factor alpha, *DHEA-S* Dehydroepiandrosterone, *BMI* Body Mass index, *WC* Waist Circumference, *BP* Blood Pressure, *HR* Heart Rate. ^a^Three samples will be collected in office during a 90-min participant appointment. Using a saliva collection aid, each participant will be given a 2-min time window to fill as much of one 2 ml cryovial as possible. If the participant fills the cryovial in less than 2 min, the time to completion will be noted on the vial. The first sample will be discarded and the other two will be sent to Salimetrics for analysis. ^b^For DHEA-S, the two samples will be pooled for analysis. The second saliva sample will be used for the analysis of all other biomarkers. The weight of the sample will be used to adjust for flow rate

### Sample size

A sample size calculation could not be estimated given the interventions proposed are novel for the population under study (Indigenous and non-Indigenous adults living in a small city in a rural area who engage in one or more cancer-risk behaviours). The sample size was selected in consultation with facilitators for the interventions, who indicated that 20–25 participants would be an effective group size to work with, and recognizing that each intervention must be run separately for males and females in keeping with cultural protocols recommended by Indigenous Elders and stakeholders.

### Statistical analysis

For prediction of cancer-risk behaviour at 1 and 6 months post-intervention, participants will be included in the analysis if they have completed 50% of the intervention they are assigned to and have completed follow-up data collection for the specific time point. We will utilize a repeated-measure, mixed-model approach in order to identify significant changes in the main outcome variables between the four groups, stratified by gender and Indigenous/non-Indigenous status. Differences in baseline PTSD symptoms severity will be considered to acknowledge nonlinear treatment effects between participants at t_1_. Potential confounding variables, such as exposure to potentially traumatizing events during the course of the intervention, will be used as covariates. Pearson’s Chi-square test and Fisher’s exact test will be used to assess the relationship between baseline characteristics and non-participation in the intervention and post-intervention data collection time points. Missing data will be handled using listwise deletion. All analyses will be performed using STATA and R statistics. The anonymized quantitative dataset analyzed during the current study will be available upon reasonable request to Dr. Cheryl Currie between Jan 1, 2023 and Dec 31, 2025.

### Data security and management

Microsoft Access is being used to manage and track participants using unique IDs assigned to each participant when they enroll in the study. Only the study team has access to this password protected database stored on a secure University of Lethbridge computer drive accessible only to the team. The questionnaire package is completed by participants during in-person data collection appointments using an iPad and a secure account with Qualtrics Inc. Qualtrics complies with the EU-U.S. Privacy Shield policies. All team members have signed confidentiality agreements. Signed consent forms are kept in a locked cabinet inside a locked room at the University of Lethbridge, separate from the master list linking participant identifiers to participant names, which will only be accessible by the PI. Data will be maintained for 10 years, and then shredded or deleted. Participants are informed of study risks in the consent form. Data monitoring will be the responsibility of the investigators.

### Safety measures

All interventions will include a licensed and experienced counsellor present in the sessions. Counsellors will regularly check in with the Primary Investigator and each other across the course of the study. Significant issues will be brought to the attention of the Health Research Ethics Board of Alberta Cancer Committee. All participants will receive a brochure created for this project that outlines all available mental health resources available for the participant.

## Ethics and dissemination

This study has received ethics approval from the Health Research Ethics Board of Alberta Cancer Committee; important protocol modifications will be reviewed by this Committee. The trial will be conducted in compliance with this study protocol, the Tri-Council Policy Statement: Ethical Conduct for Research Involving Humans (TCPS 2) and Good Clinical Practice (GCP) [58]. Findings will be disseminated at regional and international conferences, in peer-reviewed journals, and through popular media. The PI will ensure that authors on manuscripts meet authorship criteria as defined by the International Committee of Medical Journal Editors. Professional medical writers will not be used in this study.

### Current trial status

Recruitment of participants started in April 2019. Primary data analysis will begin Spring 2020. The last participant is expected to reach the primary endpoint in late 2021.

## Discussion

Social trauma is a common experience and may be a key driver of cancer-risk behaviours, particularly within Indigenous populations given the legacy of colonization. Randomized trials designed to assess the effectiveness of trauma-informed interventions that may reduce engagement in these behaviours within community-based populations are needed. This controlled outcome trial will examine trauma-informed psychotherapeutic and body-based interventions for cancer-risk behaviour. These interventions were developed through extensive consultation with expert and public stakeholders. We will oversample Indigenous adults, with stratified analyses planned specifically for this population. Limitations include the inability to blind participants due to the nature of the intervention, and the potential for limited generalizability given all data will be collected from participants living in a small city in western Canada.

## Data Availability

The dataset that will be analyzed for this study is available from the corresponding author on reasonable request.

## Abbreviations

ACEs: Adverse childhood experiences
AUDIT: Alcohol Use Disorders Identification Test
BDE-S: Brief Dissociative Experiences Scale
BGQ: Brief Grief Questionnaire
BMI: Body mass index
CRP: C-reactive protein
DBP: Diastolic blood pressure
DHEA-S: Dehydroepiandrosterone sulfate
DUDIT: Drug Use Disorders Identification Test
EOD: Experiences of discrimination
GCP: Good Clinical Practice
HLS: Historical Loss Scale
HPA: Hypothalamic-pituitary-adrenal
HR: Heart rate
IDC: Integrative drum circles
IPAQ: International Physical Activity Questionnaire
PSQI: Pittsburgh Sleep Quality Index
PTSD: post-traumatic stress disorder
RSES: Rosenberg Self Esteem Scale
SBP: Systolic blood pressure
SCS-SF: Self-Compassion Scale – Short form
SSQ: Social Support Questionnaire
TCPS 2: Tri-Council Policy Statement: Ethical Conduct for Research Involving Humans
TIY: Trauma-informed yoga
TNF α: Tumor necrosis factor alpha
VI: Vancouver Index
WC: Waist circumference
